# Sinapic acid attenuates muscle atrophy in streptozotocin-induced diabetic mice

**DOI:** 10.22038/IJBMS.2021.60324.13370

**Published:** 2021-12

**Authors:** Liu Xianchu, Liu Ming, Cheng Changhao, Deng Beiwang, Xie Jingtao

**Affiliations:** 1 Institute of Physical Culture, Hunan University of Arts and Science, 415000 Changde, China; 2 Faculty of Science, College of Furong, Hunan University of Arts and Science, 415000 Changde, China; 3 The First Affiliated Hospital of Hunan University of Chinese Medicine, 410021 Changsha, China

**Keywords:** Apoptosis, Endoplasmic reticulum- stress, Mitochondrion, Muscle atrophy, Sinapic acid

## Abstract

**Objective(s):**

Diabetes is fundamentally connected with the inability of skeletal muscle. Sinapic acid (SA) has multiple biologic functions and is diffusely utilized in diabetic complications. The purpose of this study was to explore the potential improvement effect and mechanisms of SA in streptozotocin (STZ)-induced diabetic muscle atrophy.

**Materials and Methods:**

The model of diabetic mice was established by intraperitoneal STZ (200 mg/kg) to evaluate the treatment effect of SA (40 mg/kg/d for 8 weeks) on muscle atrophy. Muscle fiber size was assessed by Hematoxylin and Eosin (HE) staining. Muscle force was measured by a dynamometer. Biochemical parameters were tested by using corresponding commercial kits. The expressions of Atrogin-1, MuRF-1, nuclear respiratory factor 1 (NRF-1), peroxisome proliferative activated receptor gamma coactivator 1 alpha (PGC-1α), CHOP, GRP-78, BAX, and BCL-2 were detected by Western blot.

**Results:**

Our data demonstrated that SA increased fiber size and weight of gastrocnemius, and enhanced grip strength to alleviate diabetes-induced muscle atrophy. In serum, SA restrained creatine kinase (CK), lactate dehydrogenase (LDH), malondialdehyde (MDA), tumor necrosis factor (TNF-a), and interleukin 6 (IL-6) levels, while enhancing total anti-oxidant capacity (T-AOC), superoxide dismutase (SOD) and catalase (CAT) levels to improve muscle injury. In gastrocnemius, SA promoted NRF-1, PGC-1α, and BCL-2 expressions, while inhibiting Atrogin-1, MuRF-1, CHOP, GRP-87, and BAX expressions.

**Conclusion:**

SA protected against diabetes-induced gastrocnemius injury via improvement of mitochondrial function, endoplasmic reticulum (ER) stress, and apoptosis, and could be developed to prevent and treat diabetic muscle atrophy.

## Introduction

Diabetes mellitus (DM) is one of the severe metabolic diseases caused by biological dysfunction of insulin. It has become a serious health concern due to its significance and continuously increasing incidence (1). Clinically, altered glucose homeostasis is the main reason for the development of diabetes. Long-term hyperglycemia is harmful to multiple tissues and organs, which results in diabetic complications, such as musculoskeletal abnormalities (2). Diabetes is a lifelong disease and severely affects patients with health and living quality (3). The mechanism of tissue and organ damages is complex in diabetes. Nevertheless, endoplasmic reticulum stress, mitochondrial malfunction, and apoptotic are the major contributors to the pathogenesis of diabetic complications (4, 5).

The ubiquitin-proteasome system (UPS), as an important proteolytic system, is involved in maintaining the balance of anabolism and catabolism during the muscle-wasting processes (6). In skeletal muscles, diabetes was proven to cause UPS dysfunction. Atrogin-1 and MuRF-1 are important indicators of the proteolytic system, which are negative regulators of muscle health by regulating proteasome-mediated target protein degradation (7). Excessive expressions of Atrogin-1 and MuRF-1 mean UPS dysfunction. In other words, diabetes evokes muscle atrophy by enhancing Atrogin-1 and MuRF-1 expressions (8). Additionally, several experimental types of research showed that suppression of Atrogin-1 and MuRF-1 expression was an effective way to improve muscle atrophy in diabetes (9). 

Sinapic acid (SA) is widespread in plants and has become a common food additive and nutraceutical in human dietary sources (10). SA is also considered a natural herbal with innumerable pharmacological benefits and used as a Chinese traditional remedy in many diseases, such as diabetes and memory deficits (11). It was observed that SA possessed anti-hyperglycemic efficacy by ameliorating insulin expression of β-cells (12). In soleus muscle, SA could powerfully enhance glucose uptake by elevating GLUT 4 levels in diabetic rats (13). In addition, SA was reported to alleviate STZ-induced diabetic complications by regulation of oxidative stress, inflammatory and apoptotic (14-16). However, there is no research on the improvement of SA on diabetic myopathy. In this study, the hypothesis of ameliorating muscular atrophy by SA in STZ-induced diabetes was proposed. Hence, we aimed to contribute to the protective effect and molecular mechanisms of SA in diabetic muscle atrophy.

## Materials and Methods

***Animals***

Male ICR mice (n=60, 8 weeks old) were obtained from Hunan SJA Laboratory Animals (Changsha, China). The weights of mice were 20±2 g. Mice were housed in a light-dark (12:12 hr) cycle with humidity (50±10 %) and temperature (23±2 ^°^C). During the treatment period, experimental mice were given free access to a standard chow diet and water. In this study, animal experiments were inspected by the Ethics Committee of Hunan University of Arts and Science (No. HUAS-2021-TY-133).

***Chemicals and reagents***

Sinapic acid (purity: ≥98%) and streptozotocin were obtained from Sangon Biotech (Shanghai, China). CHOP, BAX, and BCL-2 antibodies were obtained from Proteintech (Wuhan, China). Atrogin-1, MuRF-1, and GRP-78 antibodies were obtained from Sangon Biotech (Shanghai, China). The assay kits of CK, LDH, T-AOC, SOD, CAT, and MDA were purchased from Nanjing Jiancheng Biotechnology Institute (Nanjing, China). The detection kits of TNF-α and IL-6 were purchased from MultiSciences (Lianke) Biotechnology Co., Ltd. (Hangzhou, China).

***Experimental design***

Experimental mice were assigned to the control group (CON group, n=20), diabetes mellitus group (DM group, n=20), and sinapic acid treatment group (DM + SA group, n=20). The diabetes model was established through intraperitoneal injection of STZ (200 mg/kg). Clinical manifestations of the diabetes mellitus model were recorded. To estimate whether the DM model was successfully induced, blood samples were obtained from the caudal vein to detect glucose concentration. Mice with glucose concentration >16.7 mmol/l were deemed an applicable diabetic model. In the diabetic model, mice were intragastrically administrated with SA (40 mg/kg/day for 8 weeks), which was defined as DM+SA group. The Control group was administered an equivalent amount of saline.

***Grip strength***

Muscle force was analyzed by a dynamometer (YLS13, Anhui Zhenghua Bioinstrumentation). In the grip strength test, mice tightly gripped the stick with all limbs and pulled backward. Then, peak grip strength was observed and recorded. The experimental value was repeated three times in each mouse to calculate the average value.

***Sample preparation***

After measurement of grip strength, the experimental mouse was euthanized via injection of pentobarbital. Blood samples were collected and centrifuged to acquire serum for biochemical assessment. Gastrocnemius was weighed. One part of skeletal muscle was preserved at –80 ^°^C to evaluate the protein expression level. The remaining tissue was stored in 4% paraformaldehyde for analysis of fiber size.

***Biochemical assessment***

CK and LDH were determined to evaluate muscle injury. CK can catalyze creatine and adenosine triphosphate to form creatine phosphate and adenosine diphosphate. The CK activity can be calculated according to the amount of generated inorganic phosphorus. The detection absorbance was recorded at 660 nm. The activity of CK was shown as U/ml.

LDH as an important enzyme is involved in energy metabolism. LDH can transform lactic acid to pyruvic acid, which reacts with 2, 4-dinitrophenylhydrazine to produce pyruvate dinitrophenylhydrazone. The detection absorbance was recorded at 440 nm. The activity of LDH was shown as U/ml.

T-AOC content was measured by an spectrophotometer. In serum, many anti-oxidant substances can transform Fe^3+ ^to Fe^2+^, which reacts with phenanthroline to produce a stable complex compound. The absorbance of the reaction product was recorded at 520 nm. The level of T-AOC was shown as U/ml.

SOD activity in serum was measured by the hydroxylamine method. During the oxidation of xanthine, SOD can reduce nitrite concentration. The detection absorbance was recorded at 550 nm. The activity of SOD was shown as U/ml.

CAT activity in serum was measured by the ammonium molybdate method to assess hydrogen peroxide destruction. CAT can decompose hydrogen peroxide, but ammonium molybdate can prevent this chemical reaction. In addition, hydrogen peroxide reacts with ammonium molybdate to produce a new complex compound, the absorption of which is 405 nm. The activity of CAT was shown as U/ml.

MDA level in serum was measured by the TBA fluore-

scence method. The condensation of MDA with TBA forms a colored compound, the absorption of which is 532 nm. The level of MDA was shown as nmol/ml.

***ELISA***

TNF-a and IL-6 were determined to evaluate inflammatory response. The levels of TNF-a and IL-6 were analyzed by ELISA. Firstly, 10 μl of sample and 90 μl of detection buffer were added to coated wells. Then, 50 μl of detection antibody was added to incubate for 1.5 hr at room temperature. After washing 6 times, 100 μl of horseradish peroxidase-labeled streptavidin was added to incubate for 30 min at room temperature. In the next step, the coated wells were again washed 6 times and 100 μl of TMB was combined for 5 min. Finally, 100 μl of stop buffer was added for detection absorbance at 450 nm.

***Histological analysis***

Hematoxylin and Eosin (HE) staining was used to observe the features of skeletal muscles. After fixation with paraformaldehyde, the gastrocnemius was dehydrated with different concentrations of alcohol. Xylene was regarded as a transparency agent. Then, the gastrocnemius was embedded in paraffin. A rotary microtome was used to cut gastrocnemius into 5 um. The pathological section was stained with HE. The staining was captured under a light microscope for assessment of gastrocnemius histomorphology. The myocyte cross-sectional areas were subsequently measured and calculated using the Image J software package.

***Western blot***

Gastrocnemius was homogenized with lysis buffer and proteinase inhibitors. Protein levels were tested via the BCA method. SDS-PAGE was used to separate individual proteins, which were transferred onto the PVDF membrane by wet electroblotting. In the PVDF membrane, 5% milk sealant was used to block the nonspecific binding site. The primary antibodies were added to combine specific binding. After washing 3 times, the PVDF membrane was incubated with corresponding HRP-conjugated antibodies. The PVDF membrane was again washed 3 times and ECL chemiluminescence was combined. The protein band was captured under the imaging system. The density was normalized to β-actin and subsequently analyzed to appraise the relative ratio of protein expressions.

***Statistics***

All data were shown as mean±SD. The results were analyzed with SPSS 16.0 software. Statistical difference was demonstrated by a one-way ANOVA test. *P*<0.05 was deemed statistically significant.

## Results

***Effects of SA on diabetes-induced skeletal muscle atrophy***

**Figure 1:**
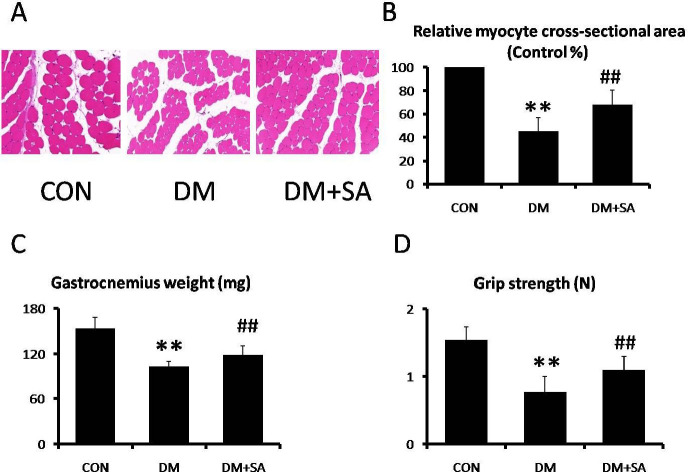
Effects of Sinapic acid (SA) on diabetes-induced skeletal muscle atrophy. (A, B) Hematoxylin and Eosin (HE) staining was utilized to analyze morphological characteristics in gastrocnemius (200 X). (C) Weight of gastrocnemius. (D) Grip strength of skeletal muscle. Data are presented as mean ± SD. n=10 per group. **P<0.01 compared with CON group; ##*P*<0.01 compared with diabetes mellitus (DM) group

HE was utilized to evaluate the ameliorations of SA against diabetes-induced morphological changes in skeletal muscles. Figures 1A and B show representative myocyte cross-sections were remarkably decreased in the DM group (*P*<0.01), while SA dramatically increased the fiber size in the gastrocnemius (*P*<0.01). Figures 1C and D show gastrocnemius weight and grip strength were markedly decreased in the DM group (*P*<0.01), while SA observably reversed these changes (*P*<0.01). 

***Effects of SA on CK and LDH in serum***

**Figure 2:**
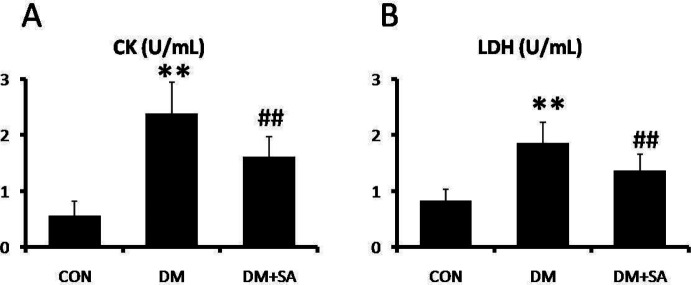
Effects of Sinapic acid (SA) on creatine kinase (CK) and lactate dehydrogenase (LDH) in serum. A spectrophotometer was utilized to examine (A) CK and (B) LDH levels. Data are presented as mean ± SD. n=10 per group. ** *P*<0.01 compared with CON group; ## *P*<0.01 compared with diabetes mellitus (DM) group

To evaluate the regulation of SA on diabetes-induced muscle injury, the activities of CK and LDH were examined in serum. As shown in Figure 2, CK and LDH activities were remarkably elevated in the DM group (*P*<0.01), while SA observably decreased CK and LDH activities to relieve muscle injury (*P*<0.01).

***Effects of SA on T-AOC, SOD, CAT, and MDA in serum***

**Figure 3:**
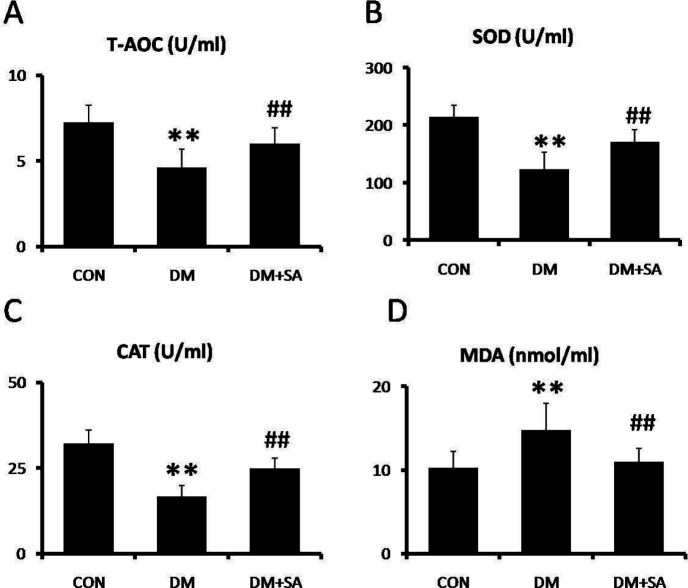
Effects of Sinapic acid (SA) on anti-oxidation in serum. A spectrophotometer was utilized to examine (A) total anti-oxidant capacity (T-AOC), (B) superoxide dismutase (SOD), (C) catalase (CAT), and (D) malondialdehyde (MDA) levels. Data are presented as mean ± SD. n=10 per group. ** *P*<0.01 compared with CON group; ##*P*<0.01 compared with diabetes mellitus (DM) group

To evaluate anti-oxidation of SA in diabetes-induced muscle injury, the levels of T-AOC, SOD, CAT, and MDA were examined in serum. As shown in Figure 3, T-AOC, SOD, and CAT levels were remarkably reduced, while MDA levels were remarkably elevated in the DM group (*P*<0.01). In contrast, SA observably reversed these changes to alleviate oxidative damage (*P*<0.01).

***Effects of SA on TNF-a and IL-6 in serum***

**Figure 4:**
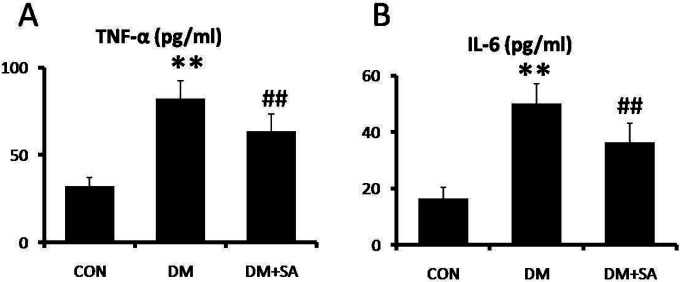
Anti-inflammatory effects of Sinapic acid (SA) in serum. ELISA was utilized to examine (A) tumor necrosis factor (TNF-a) and (B) interleukin 6 (IL-6) levels. Data are presented as mean ± SD. n=10 per group. ** *P*<0.01 compared with CON group; ## *P*<0.01 compared with diabetes mellitus (DM) group

To evaluate the anti-inflammatory effects of SA on diabetes-induced muscle injury, the concentrations of TNF-a and IL-6 were examined in serum. As shown in Figure 4, TNF-a and IL-6 concentrations were remarkably elevated in the DM group (*P*<0.01), while SA observably reduced TNF-a and IL-6 concentrations to alleviate inflammatory stimulation (*P*<0.01).

***Effects of SA on muscle-specific E3 ubiquitin ligases in gastrocnemius***

**Figure 5:**
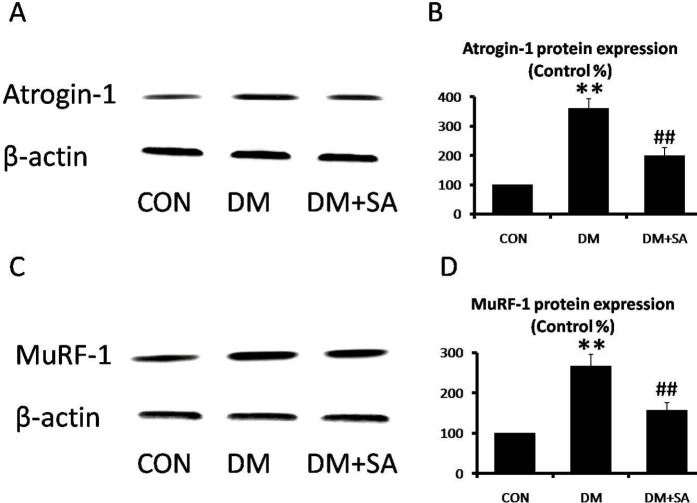
Effects of Sinapic acid (SA) on muscle-specific E3 ubiquitin ligases in gastrocnemius. (A, B) Western blot was utilized to examine Atrogin-1 and MuRF-1 levels (C, D) Quantification of Atrogin-1 and MuRF-1 expressions. Data are presented as mean ± SD. n=3 per group. **P<0.01 compared with CON group; ## *P*<0.01 compared with diabetes mellitus (DM) group

To evaluate the modulation of SA in the diabetes-induced ubiquitin-proteasome pathway, Atrogin-1 and MuRF-1 expressions were examined in gastrocnemius. As shown in Figure 5, expressions of Atrogin-1 and MuRF-1 were remarkably elevated in the DM group (*P*<0.01), while SA observably decreased these muscle-specific E3 ubiquitin ligases to relieve protein degradation (*P*<0.01).

***Effects of SA on mitochondrial biology in gastrocnemius***

**Figure 6:**
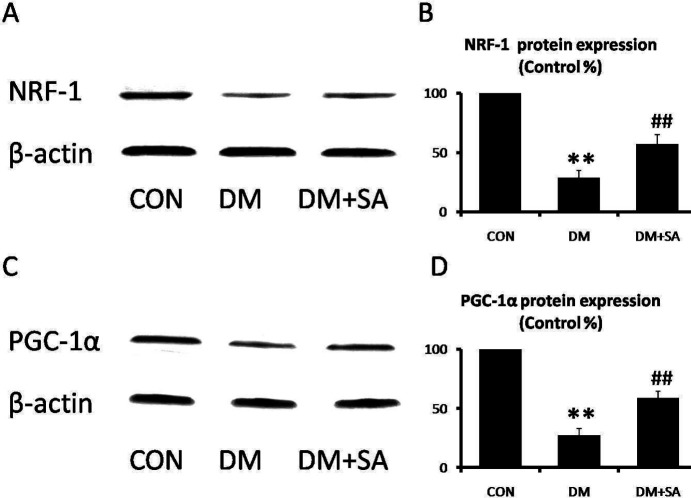
Effects of Sinapic acid (SA) on mitochondrial biology in gastrocnemius. (A, B) Western blot was utilized to examine NRF-1 and PGC-1α levels. (C, D) Quantification of NRF-1 and PGC-1α expressions. Data are presented as mean ± SD. n=3 per group. ** *P*<0.01 compared with CON group; # *P*<0.05, ## *P*<0.01 compared with diabetes mellitus (DM) group

NRF-1 and PGC-1α are involved in the regulation of mitochondrial function. As shown in Figure 6, expressions of NRF-1 and PGC-1α were remarkably decreased in the DM group (*P*<0.01), while SA observably increased these expressions to improve mitochondrial dysfunction (*P*<0.01).

***Effects of SA on ER stress in gastrocnemius***

**Figure 7:**
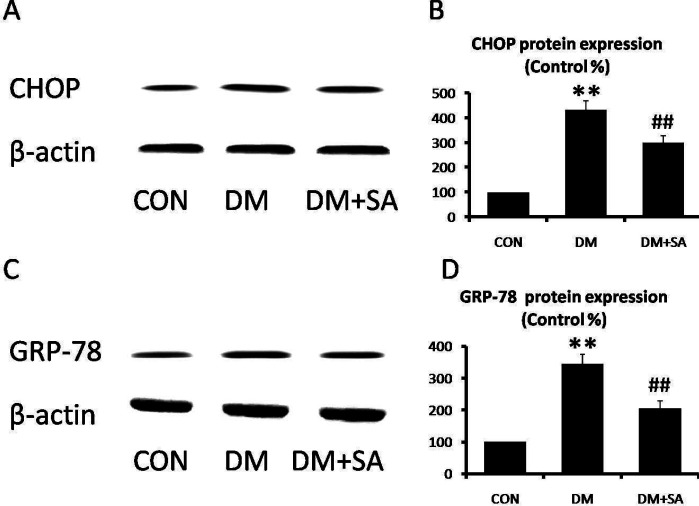
Effects of Sinapic acid (SA) on endoplasmic reticulum (ER) stress in gastrocnemius. (A, B) Western blot was utilized to examine CHOP and GRP-78 levels. (C, D) Quantifications of CHOP and GRP-78 expressions. Data are presented as mean ± SD. n=3 per group. **P<0.01 compared with CON group; ##*P*<0.01 compared with diabetes mellitus (DM) group

CHOP and GRP-78 are well-known and reliable indicators of ER stress. As shown in Figure 7, expressions of CHOP and GRP-78 were remarkably elevated in the DM group (*P*<0.01), while SA observably reversed diabetes-induced enhancement of ER stress markers (*P*<0.01).

***Effects of SA on apoptosis in gastrocnemius***

**Figure 8:**
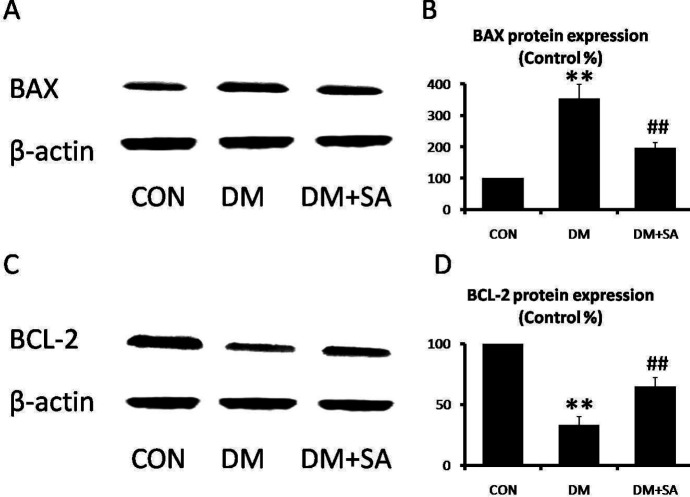
Effects of Sinapic acid (SA) on apoptosis in gastrocnemius. (A, B) Western blot was utilized to examine BAX and BCL-2 levels. (C, D) Quantification of Bax and Bcl-2 expressions. Data are presented as mean ± SD. n = 3 per group. **P<0.01 compared with CON group; ##*P*<0.01 compared with diabetes mellitus (DM) group

BAX and BCL-2 are vital markers of apoptosis, which were linked to cellular damages. As shown in Figure 8, BAX expression was remarkably elevated in the DM group, while SA observably inhibited diabetes-induced BAX levels in gastrocnemius (*P*<0.01). However, BCL-2 expression was remarkably suppressed in the DM group, while SA observably elevated BCL-2 level in gastrocnemius (*P*<0.01).

## Discussion

Skeletal muscle is not only a locomotive organ but also an endocrine organ. Plentiful bioactive molecules are synthesized and secreted in skeletal muscle (17). In hyperglycemia-induced metabolic diseases, skeletal muscle is involved in glucose storage and glucose uptake (18). Myopathy is one of the diabetic complications induced by altered glucose homeostasis and easily causes motor disturbance (19, 20). The muscle tissue suffers a series of lesions and adverse changes during diabetic disorders. For example, altered glucose homeostasis results in loss of muscle mass, reduction of fiber size, and impairment of force generation (21). In a sense, intervention of diabetic myopathy has been predicted to ameliorate hyperglycemia-induced metabolic dysfunction. In this study, our results showed SA elevated skeletal muscle weight and fiber size along with increased grip strength in STZ-induced diabetic mice. In addition, excessive levels of CK and LDH are released into the bloodstream because of hyperglycemia-evoked muscle injury. SA was demonstrated to relieve CK and LDH levels, which was in accord with amelioration of muscle injury in diabetes. The above results elucidated that SA was effective in treatment and improvement of diabetic myopathy.

Muscle atrophy is a widespread disorder that leads to progressive deterioration of exercise performance (22). Diabetes plays an important role in muscle atrophy. Proteasomal protein degradation which contributes to loss of muscle mass is a considerable phenomenon in muscle atrophy (23). In diabetes, hyperglycemia induces protein degradation, and the increased production of ubiquitin ligase promotes protein catabolism of skeletal muscle. Atrogin-1 and MuRF-1 are specific E3 ubiquitin ligases in muscle. In animal models, atrogin-1 and MuRF-1 expressions were increased to stimulate muscle atrophy by the ubiquitin-proteasome pathway (24). Previous studies also showed hyperglycemia-induced protein degradation, which was characterized by enhancing atrogin-1 and MuRF-1 expressions, was closely relevant to muscle atrophy (25). In this study, our results showed SA reduced induction of atrogin-1 and MuRF-1 expression in gastrocnemius of STZ-induced diabetes. Hence, SA was effective in relieving proteasomal protein degradation induced by hyperglycemia.

Mitochondrion is a main organelle of ATP product. Exercise is an effective way to prevent muscle atrophy. It consumes a lot of energy during executing the physiological function of skeletal muscle (26). Hence, mitochondrial dysfunction is associated with muscle atrophy. In addition, many studies have reported that hyperglycemia plays an important role in the pathogenesis of mitochondrial dysfunction (27, 28). NRF-1 and PGC-1a are involved in the regulation of mitochondrial biogenesis. NRF-1 and PGC-1a have been observed to be reduced in hyperglycemia-induced skeletal muscle damage (29). Therefore, muscle atrophy is particularly susceptible to mitochondrial dysfunction (30). More importantly, SA could deplete mitochondrial enzymes in ISO-induced myocardial injury (31). Our findings demonstrate SA elevated NRF-1 and PGC-1a expressions in gastrocnemius of STZ-induced diabetes, suggesting SA could safeguard skeletal muscle mitochondria from damage.

Endoplasmic reticulum stress is triggered by altered glucose homeostasis, leading in turn to diabetic complications (32). CHOP and GRP-78 are ER stress markers as reported to be increased in diabetic myopathy (33). Initially, ER is involved in protein modification, but stressful stimuli result in unfolded protein response to trigger muscle atrophy (34, 35). We detected CHOP and GRP-78 expressions in gastrocnemius and found the expected rise in STZ-induced diabetes indicating the occurrence of ER stress, while these expressions were decreased in mice treated with SA. Several pieces of research have shown that reduction of ER stress is effective in mediating the pathogenesis of muscle atrophy (36). In addition, SA was also reported to attenuate ER stress in 6-OHDA-induced neurotoxicity (37). Taken together, diabetes enhanced expressions of ER stress signaling pathway in gastrocnemius, whereas SA reduced CHOP and GRP-78 expressions to restrain ER stress.

Previous research indicated that the apoptosis signaling pathway was evoked via multiple diseases such as diabetes and muscle atrophy (38, 39). BAX and BCL-2 are linked to apoptotic signaling pathways, abnormal expression of which are known to contribute to muscle atrophy occurrence (40, 41). Previous research reported SA possessed anti-apoptotic activity against diabetic nephropathy via suppression of BAX expression and increase of BCL-2 expression (14). In addition, mitochondrial dysfunction and ER stress promote the proteasomal protein degradation of skeletal muscle to cause serious cellular damages and induce apoptosis. Therefore, we hypothesized that the defensive function of SA on diabetic muscle atrophy was mediated by inhibiting the activation of apoptosis. In this study, our results showed SA inhibited apoptosis in gastrocnemius, as represented by down-regulating BAX expression and up-regulating BCL-2 expression, to relieve diabetic muscle atrophy.

## Conclusion

Our research indicated that SA improved hyperglycemia-induced muscle atrophy, which was associated with its modulation of mitochondrial function, moderation of ER stress, and suppression of apoptosis. The above results demonstrated SA possessed various biological effects and might be a promising candidate for relieving muscle atrophy in diabetes.

## Authors’ Contributions

LXC and LM contributed to the conception and design of the study. LM carried out histological evaluations and Western blot analysis, LXC, CC, DB, and XJT carried out all other experiments and contributed to the interpretation of the results. LM drafted the manuscript and revised the final manuscript critically for publication in the journal. The corresponding author declares that all listed authors meet the authorship criteria and that no other authors meeting the criteria have been omitted.

## Conflicts of Interest

No conflicts of interest are associated with this work.
